# 

**Published:** 1804-12-01

**Authors:** 


### To CORRESPONDENTS.

Mr. Chamberlaine's work on Worms and Vermifuges will be noticed in our next Number.

Communications are received from Drs. Adams, Turner, and Kinglake, Messrs. Hall, Simmons, Low, Davies, Crowfoot, Whitlam, Ryal, Bond, Chamberlaine, Peaal, Shearly, Williams, Philologus, T. R. Humanus, and H.

---

### ERRATA.

P. 448, l. 12, for Pazzoni, read Pezzoni.
459, l. 21, for Weeada, read Weende.
463, l. 1, for Docteorem, read Doctorem.

END OF VOL. XII.

### INDEX.

ABDOMEN, cafe of dropfical enlargement of the 249
Abercrombie, General, on the death wound of 173, 243, 421
Abernethy's furgical obfervations reviewed 176, 266
——— lectures announced 285
Academicus in reply to Mr. Cuming 73
Acid, difcovery of a new vegetable 92
—— method of preparing gallic 282
Adams, Dr. on Gout 141
——— on cutaneous eruptions 193
——— on the cow-pox, remarks on 332
——— on hydatids 552
Advertifements, fingular quack 138
Agaric, defcription of the 512
Ague, Mr. Davie on 499
Agues, on the cure of 430
Air, on heat difengaged by compreffion of 575
Air pump vapour bath, on the 66
Alder, Mr. on the difordered ftate of the lungs 238
—— his treatifes on fever, announced 288
—— botanical defcription of 35, 223
Ammoniated iron, preparation of 192
Anecdotes, medical 427
Anemometer, invention of an 283
Aneurifm of the aorta, cafe of 235
——— cafes of 275
Angelica, botanical defcription of 364, 5
Animal heat in typhus 244
——— jelly in intermittents, on 431
Animals, on the falivary glands in 548
Anus, cafe of imperforated 236
Aorta, aneurifm of the 235
Apparatus, account of a fteam 516
Apothecaries, frauds of 429
——— their number in London 430
Armiger's, Mr. demonftrations announced 384
Arfenic, its effects on men and horfes *(note)* 278
Artery, wounded brachial 339
Afcites, on digitalis in 116
Afia, account of vaccine innoculation in 122, 447
Affifanders, botanical defcr'ption of 554
Afthma, on digitalis in 115
Athens, introduction of vaccination at 449
Atkinfon's, Mr. cafe of monftrofity 13
——— obfervations on a fevere dyfentery 17

Atrophy of the tefticles, on an 518
Babington's, Dr. lectures announced 284
Badham's, Dr. lectures announced 287
Bardfley's, Dr. cafe of poifon from a vegetable fungus 385, 512
——— on two cafes of fuppofed rabies canina 387
Bark, on the, in gout 141
—— on the febrifuge fubftance in the 430
Bartlett, Mr. on opium in intermittent fever 3
——— on nicotiana 401
Batty's Dr. lectures announced 286
Beddoes, Dr. on the influenza and the preventive medical inftitution 479
Bernard, T. efq. on the fever inftitution 163
Bindweed, defcription and properties of 129
Birch tree, defcription and properties of the 34, 36
Blake's, Mr. defcription of an improved tourniquet *(with a plate)* 336
Blegborough, Dr. on the gout 63, 375
——— on animal heat in typhus 244
——— reply to 341
Blennorhœa, on the inoculation of 519
Blizard's, Meffrs. lectures announced 384
Bones, on preparing broth from 350
——, on the veins of the 551
Books, on reading medical 506
BOOKS, NEW MEDICAL ANNOUNCED. Donovan's Natural Hiftory of Britifh Shells, 96. Dr. Patterfon on the Climate of Ireland, 192. Cuvier on extinct Animals, *ib.* De Villeneuve's Defcription of Plants at Ghent, *ib.* Mr. Alder on Fevers, 288. Dr. Beddoes's Report of the Proceedings of the Preventive Medical Inftitution, 479. Mr. Edlin's Treatife on Bread Making, *ib.*
BOOKS, CRITICAL ANALYSIS OF NEW MEDICAL.—Obfervations on Schirrhous Tumours and Cancers of the Breaft, by J. Nooth, 75. Cafes of Small-pox fubfequent to Vaccination, by W. Goldfon, 79. Dr. Trotter's Effay on Drunkennefs, 86. A Treatife on Gun-fhot Wounds, by T. Chevalier, 174. Surgical Obfervations, by J. Abernethy, 176, 266. Account of a new Medicine for Gout, by A. Welles, 180. An Anfwer to Mr. Goldfon, by John Ring, 181. Medical Reports of Cafes of Inoculation and

Re-Inoculation with Variolous and Vaccine Matter, by J. Rollo, M. D. 263. Experiments on Vaccination, by R. Dunning, 264. Effay on the Advantages of the Flexible Metallic Bougies, by W. Smyth, 276. A complete Syftem of Veterinary Medicine, by J. White, 278 Appendix to Practical Obfervations on the Venereal Difeafe, by E. Geoghegan, 279. Schola Medicinæ, or the New Univerfal Hiftory and School of Medicine, by W. Rowley, M. D. 469 Experiments on Vacciolation, by S. Hill, 473. A Differtation on Gout, by R. Kinglake, M. D. 561. A Medical Gloffary, by W. Turton, M. D. 565. Pharmacopœia Medici Practici Univerfalis, &c. auctore F. Swediaur, M. D. *ib.*
Borage, defcription and virtues of 126
Botanical defcription of Britifh plants, 38, 124, 220, 361, 554
Box, botanical defcription of the 37
Bougies, on metallic 276
Brachial artery, cafe of a wounded 339
Bradley's, Dr. lectures announced 287, 384
———— cafe of fudden death 391
Brain, cafe of fudden death from a fuppofed morbid affection of the 391
Brainular affection, cafe of 109
———— Dr. Patterfon's cafe of 196
Bread making, propofed treatife on 479
Brody's, Mr. cafes in furgery *(with a plate)* 513
Bronchocele, on the 9
Brookes's, Mr. lectures announced 286
Broth, on preparing it from bones 350
Brown's Dr. lectures announced 286
Bubo, obfervations on 279
Buchholz's new method of preparing emetic tartar 189
———— experiments on mercurius dulcis 574
Buckbean, defcription and virtues of 127
Buckthorn, defcription and qualities of 221
Burdock, defcription of the 231

Cancer, on 410
Cancers in the breaft, on the treatment of 75
Canine madnefs, on 513
Caries of the os frontis, cafe of 394
Carlifle's cafe of ftrangulated hernia 337
———— Mr. lectures announced 285
Carpue's, Mr. lectures announced 288
Carraway, botanical defcription of 555
Carrot, wild, defcription of the 231
Cafes of fmall-pox fubfequent to vaccination reviewed 79
Catarrhs, on chronic 258
Catarrhus fuffocativus, on cafes of 409
Centory, defcription and qualities of 220
Cerebellum, lofs of a portion of the 15
Chalybeate fpring, difcovery of a 240
Charcoal ufed in filtering water 96
———— on various ufes of 283
Chervil, botanical defcription of 370
Chevalier, Mr. on gun fhot wounds, reviewed 174
———— his lectures announced 288
———— obfervations on his cafes of catarrhus 409
Chicken pox, on the 537
Children, on the difeafes of 51
Chinefe radifh, cultivation of 93
Cholera morbus, on the treatment of 102, 219, 262
Clark's, Dr. defcription of improved furgical inftruments *(with a plate)* 544
Clarke's, Dr. lectures announced 287
———— cafe of fudden death 391
Claveau des Moutons, on the 457
Cleghorn's, Dr. lectures announced 286
Cline's, Mr. lectures announced 284
Coleman's, Mr. Veterinary lectures announced 284
Colica pictonum, cured by nicotiana 403
Comfrey, botanical defcription of 125
Convulfions, on cold water in 508
————, cafe of 112
Cooke, Mr. on eryfipelas 523
Cooke's, Dr. lectures announced 384
Cooper's, Mr. ditto 284
Copal, on the folution of 93
Coriander, botanical defcription of 369
Correfpondents, to 96, 192, 288, 384, 576
Cortex falicis latifolia, on the 372
Cow-pock, on a proper claffic term for 335
Cow-pox, Dr. Walker on Dr. Adams's Obfervations on 333
————, Mr. Ring on 356
————, fee VACCINATION, &c.
Cowflip, botanical defcription of 127
Cow-weed, defcription of 370
Crieghton, Mr. on the efficacy of vacciola 205
Crochford, Mr. on ditto 207
Cullurne, Mr. on the Kenfington cafe 539
Cuming, Mr. on mercurial inunction in typhus 52
————, reply to, by Academicus 73
————, on digitalis in pneumonia 113
————, on vaccine prophylaxis 209
————, on leprofy 404
Cupping-glafs, on, in hydrophobia 344, 436
Currant, botanical defcription of 225
Curry's, Dr. lectures announced 284
Cutaneous eruptions, Dr. Jenner on the effects of 97
————, Dr. Adams on 193
Cuvier on extinct animals, announced 194

Davie, Mr. on ague 499
Day, Dr. account of 137
Death, cafe of fudden 391
De Carro, Dr. on vaccination 122
———— on Mr. Goldfon's pamphlet 462

[illegible]nnifon's, Dr. lectures announced 287, 384
[illegible]tition, on 108
[illegible]rrhœa, on 103, 357, 468
[illegible]igitalis, its benefits in pneumonia 113
Difeafes in an eaftern diftrict of London, from May 20 to June 20, 1804, 90
——— from June 20 to July 20 112
——— from July 20 to Auguft 20 262
——— from Auguft 20 to Sept. 20 382
——— from Sept. 20 to Oct. 20 468
——— from Oct. 20 to Nov. 20 572
Difpenfary, propofals for a new 576
Dodder, botanical defcription of the 37
Dogberry tree, defcription and qualities of 30
Donovan's, Mr. Natural Hiftory of Britifh Shells, announced 96
Douglas's, Dr. account of a chalybeate fpring 240
Dropfy, cafe of 249
Drunkennefs, effay on 86
Dunning's, Mr. experiments on vaccination 264
Dupuytren, Mr. on the veins of bones 551
———, on the larynx in eunuchs 575
Duvernoy's obfervations on the falivary glands in animals 548
Dyfentry, obfervations on fevere 17
——— on the prevalence of 468

Earth, analyfis of a new 69
Edgar's, Mr. cafe of excrefcence on the fcrotum 198
Edinburgh new difpenfatory, remarks on the 55
——— phyficians and furgeons on vaccination 11

Edlin's, Mr. Treatife on Bread Making, announced 479
Egypt, medical fketches of the expedition to 77
Elder, botanical defcription of 558
Elgin, Lord and Lady, their journey in Greece 448
Elm, common, botanical defcription of 230
Emetic tartar, method of preparing 189
Empiricifm, on quacks and 137, 212, 423
Epilepfy, on 112
Errata 96, 288, 480, 576
Eruptions, on the effects of cutaneous 97, 193
——— after vaccination, on 358
Eryngo, fea, botanical defcription of 231
Eryfipelas, on 404
———, Mr. Cooke on 523
Evans's, Mr. cafe of fracture 15
Eunuchs, on the formation of the larynx in 575
Experiments on vaccination 264
——— on muriat of foda 283
——— on vacciolation 473
Experiments on hydrargyrus muriatus mitis 574
Eye, difpenfary propofed for perfons afflicted with difeafes of the 576

Farquhar, Sir Walter, on infectious fevers in London 161
Febrifuge fubftance in bark, on the 430
Fees, on medical 427
Fennel, botanical defcription of 555
Fermentation, prize queftion on 96
Fever, on opium in intermittent 3
——— obfervations on the fcarlet 25
——— on puerperal 27
——— on the efficacy of animal jelly in 431
——— fuccefsfully treated by frictions with oil 382
——— inftitution, report of the committee on the 161
Fiedler's, Mr. method of preparing gallic acid 382
Filtering apparatus, account of a new 96
Foffil, chemical analyfis of a Swedifh 69
Fox's, Mr. lectures announced 284
Fracture of the occipital bone, cafe of 15
Frampton's lectures announced 384
Freer's, Dr. lectures announced 286
Fullwood's Rents, fuppofed cafe of a failure of vaccination in 384
———, report on the cafe of 573
Fungus, cafe of poifon from a vegetable 385, 512

Gallic acid, mode of preparing 282
Galvanifm, on its efficacy in hydrophobia 259, 513
Garnett, Dr. on gout 379
Garthfhore, Dr. on the Fever Inftitution 163
Gaftrodynia, cafe of 289
Gehlen, M. on our knowledge of opium 38, 117
Geoghegan's Obfervations on the Venereal Difeafe, reviewed 279
Gilham, Mr. on the death wound of General Abercrombie 288
Gillefpie's, Mr. ftatement of the treatment of prifoners at Norman Crofs 345
Glands falivary, in animals, on 548
Glafgow, medical lectures and regulations in the Univerfity of 286
Glafs-wort, defcription of the prickly 229
Goldfon's pamphlet, remarks on 44, 168
——— reply to Dr. Walker 49
——— Cafes of Small-pox fubfequent to Vaccination, reviewed 79
———, anfwer to, by Mr. Ring 181
———, Dr. Macdonald's obfervations on 298
———, Dr. De Carro on 462
Gonorrhœa, on the inoculation of 519
Gout, Dr. Blegborough on the 62, 375

### INDEX.

Gout, Dr. Stenhoufe on the 66
—— on Peruvian bark in 142
—— difcovery of a new medicine for 180
—— Candidus on 216
—— Dr. Trotter on cold water in 237
——Mr. Mantal on the 500
—— review of Dr. Kinglake's Dissertation on the 561
Greenhow's, Mr. cafe of caries of the os frontis 393
Griffenberg, Dr. account of 212
Griffin, Rev. Mr. anfwer to his queries on vaccination 11
Guinea worm, obfervations on the 434
Gums, ufe of charcoal in fcorbutic 284
Gun-fhot Wounds, review of a treatife on 174
Guy's Hofpital, lectures at 284

Haighton's, Dr. lectures announced 284
Halle, Mr. on animal jelly in intermittent fevers 431
Harman and Co. their filtering apparatus defcribed 96
Harrifon, Dr. on the fheep pox 457
Hawker, Dr. on ophthalmia 407
Head-ache, inftance of fuccefsful treatment of 1
Headington's, Mr. lectures announced 384
Heart's-eafe, botanical defcription of 224
Heat disengaged by compreffion of air 575
Helfham's, Dr. cafe of hydrocephalus internus 5
Hemlock, defcription and qualities of 361
—— drop wort, defcription of 366
Henbane, botanical defcription of 132
Hennen, Mr. on fponge in ophthalmia 233
Hepatitis fuppurans, cafe of 498
Hermbftaedt, Mr. on preparation of broth from bones 350
Hernia, cafe of ftrangulated 337
Herpetic exanthemata, ufe of charcoal in 283
Higgin's, Dr. meteorological journal 381, 480
Hill, Mr. on cancer 410
——'s experiments on vaccination 473
Hippocrates, practice and character of 470
Honey, effects of charcoal on 283
Honeyfuckle, botanical defcription of 130
Hooper's, Dr. lectures announced 286
Hop, defcription and qualities of the 228
Hound's tongue, botanical defcription of the 124
Hume, Mr. on modern pharmacopœias 55
——, on the cupping glafs in hydrophobia 344
Hunold, Dr. on the ufes of charcoal 283
Hydatids, Dr. Adams on 552
Hydrocephalus internus, cafe of 5
Hydrophobia, efficacy of Galvanifm in 259
—— on the cupping glafs in 344, 436
Hydrophobia, remarks on Roffi's cafes of 387, 512
Infants, on fome confequences of weaning 418
Inftruments, defcription of improved furgical *(with engravings)* 198, 544
Intermittent fever, on opium in 3
—— efficacy of animal jelly in 431
Inteftinal canal, method of deftroying worms in the 283
Inunction, efficacy of mercurial 52, 481
Ireland, a treatife on the climate of, announced 192
Iron, mode of preparing ammoniated 192
——, acetated ethereal tincture of 569
Itch, peculiar kind of in Madeira 194
Ivy, defcription and qualities of 226
Jardine's Mr. defcription of improved furgical inftruments 198
Jeffrey's, Dr. lectures announced 286
Jenner. Dr. on the effects of cutaneous eruptions 97
—— on modification of the vaccine veficle 98
Jennerian inftitution in Suffex, account of 10, 477

Kenfington, on the cafe at 446
—— cafe, Mr. Ring on the 529
Keutfch's Dr. treatment of fevers in the Weft Indies 282
Kinglake's Dr. Treatife on the Gout, review of 561
Klaproth's, Mr. chemical analyfis of a Swedifh foffil 69
—— difcovery of a new vegetable acid 92
Krueger's, Mr. Method of obtaining concentrated vinegar 111

Laconia, introduction of the cow-pox by the inhabitants 449
Lac. fulphuris, new mode of making 111
Lady's mantle, botanical defcription and properties of 34
Lambe's, Dr. cafe of inveterate head-ache 1
Lamert, Dr. account of 425
Larynx in Eunuchs, on the formation of the 575
Larrey, Mr. on the Guinea worm 434
—— on an atrophy of the tefticles 518
—— on the inoculation of blenorrhœa 519
Lectures medical announced— Meffrs. Cline and Cooper's, on the Principles and Practice of Surgery, 284. Drs. Babington and Curry on the Practice of Medicine, *ib.* Dr. Babington and Mr. Allen, on Chemiftry and Experimental Philofophy, *ib.* Dr. Curry, on the Theory and Practice of Medicine, and Materia Medica, *ib.* Dr. Haighton, on Midwifery and on Physiology, *ib.* Mr. Fox, on the Structure and Diseases of the Teeth, *ib.* Mr. Coleman, on the Veterinary Art, 284. Messrs. Lynn, Morel, and Carlisle, on Surgical Operations, 285. Drs. Roberts and Powell, on the Theory and Practice of Medicine, &c. *ib.* Mr. Abernethy, on Anatomy, Physiology, and Surgery, *ib.* Dr. Powell, on Chemistry and the Materia Medica, *ib.* Dr. Thynne, on Midwifery and the Diseases of Women and Children, *ib.* Messrs. Wilson and Thomas, on Anatomy, Physiology, and Surgery, *ib.* Mr. Brookes on Anatomy, Physiology and Surgery, 286. Dr. Hooper, on the Practice of Physic, Materia Medica, and Pharmaceutical Chemistry, *ib.* Dr. Batty, on the Theory and Practice of Midwifery, *ib.* Lectures and Regulations in the University of Glasgow, *ib.* Dr. Bradley, on the Theory and Practice of Medicine, 287. Dr. Pearson, on Physic and Chemistry, *ib.* Dr. Badham, on the Practice of Physic, Chemistry, and Materia Medica, *ib.* Drs. Dennison and Squire, on Midwifery, and the Diseases of Women and Children, *ib.* Dr. Clarke, on Midwifery, *ib.* Mr. Syer, on Midwifery, 288. Mr. Taunton, on Anatomy, Physiology, and Surgery, *ib.* Mr. Carpue, on Anatomy, *ib.* Mr. Chevalier, on Surgery, *ib.* Messrs. Headington and Frampton, on Anatomy and Surgery, 384. Mr. Armiger's Demonstrations, *ib.* Messrs. Blizard, on Surgery, *ib.* Dr. Cooke, on the Practice of Physic, *ib.* Dr. Dennison, on Midwifery, *ib.* Dr. Bradley, on the Practice of Medicine, *ib.* Dr. Reid, on the Theory and Practice of Medicine, *ib.* Mr. Thomas, on Surgery, *ib.* Mr. Moor, on the Structure and Diseases of the Teeth, *ib.*

Leeches, on the late mortality among 219, 357

——— on the preservation and management of 485

Leese, Mr. on vaccination 164

Leeson's, Mr. case of semiosseous tumour 465

Lenticular, description of an improved 203

Leprosy, observations on 404

Lichen Islandicus, on the 463

Lobelia, on the 514

London, account of diseases in an eastern district of 9[illegible], 112, 262, 382, 468, 572

London Dispensatory, remarks on the 357

London Hospital, announcement of lectures at the 384

Lowe, Mr. on Hydrophobia 436

Lungs, on the diseased state of the 238

Macdonald, Dr. on Mr. Goldson's Pamphlet 298

——— remarks on 535

Madder, botanical description of 28

Male's, Dr. case of hepatitis suppurans 497

Malta, address to the inhabitants of 443

Mantal, Mr. on gout 500

Marshall, Dr. testimonials in favour of 443

Marsh trefoil, botanical description of 127

Master wort, common, description of 371

Mayersbach, Dr. account of 213

Medical and Physical Intelligence 93, 189, 283, 382, 477, 573

——— report of cases of inoculation and re-inoculation reviewed 263

——— lectures, see LECTURES

——— anecdotes 427

——— books, on reading 506

Medicine, on the progress of 419

Mercurial inunction in typhus, on, 52, 481

Mercurius dulcis, experiments on 574

Mercury goose-foot, botanical description of 227

Meteorological journal at Brompton 381, 480, 571

M'Gregor's Medical Sketches of the Expedition to Egypt, reviewed 77

Millett's, Mr. fatal case of diarrhœa 51

Misseltoe, description and properties of 32

Monstrosity, case of 13

Moodie's, Dr. case of extensive suppuration 252

Moor's, Mr. lectures announced 384

Mortality, bill of, in America 94, 190

Mortification, case of 483

Moxa, description of the 35

Mulberry, white, saline substance found in the bark of the 92

Mullein, description of the great white 130

Muriat of ammonia used in preparing ammoniated iron 192

Nettle, botanical description and qualities of 31

Newton, Sir Isaac, on his discovery 455 (*note*)

Nicotiana, on the virtues of 401

Nightshade, botanical description of the different species of 133

Nitre, its power in stopping mortification 483

———, on the composition of 485

Nitrous acid, its power in destroying contagion 484

Nooth's, Mr. observations on the treatment of schirrhous tumours and cancers, reviewed 75

Norman-Cross, treatment of prisoners in the hospital at 345

North, the Hon. Mr. his letter on the cow-pox 283

Notice from the Vaccine-pock Inftitution 90

Occipital bone, cafe of fracture of the 15
Oils, frictions by, a remedy for fevers in the Weft Indies 282
Ophthalmia, on the ufe of fponge in 233
——— Dr. Hawker on 407
——— of Egypt, obfervations on the 79
Opium, on its ufe in intermittent fever 3
——— on our knowledge of 38, 117
Orache, ftinking and wild, defcription of 228

Panfies, botanical defcription of 224
Paris, letter from, on Goldfon's pamphlet 348
Parfley, botanical defcription of 557
Patterfon's, Dr. cafes of brainular affection 109, 196
——— Obfervations on the climate of Ireland announced 192
——— cafe of a child 198
Pearfon's, Dr. lectures announced 287
Pellitory of the wall, defcription and qualities of 30
Peruvian bark, its ufe in gout 142
——— on the febrifuge fubftance in 436
Pharmaceutical proceffes, account of new 111
Pharmacopœias, on modern 55
Philologos, on a proper or claffical term for cow-pock 335, 517
Phimofis, cafe of 20
Pimpernel, defcription and properties of 128
Plague, remarks on the 79
——— true character of the 91
——— whether it mitigates the fmall-pox 454
Plantain, common, defcription of 29, 124
Plants, catalogue of Britifh 28, 124, 220, 361, 554
Pneumonia, on digitalis in 113
Poifon from a vegetable fungus, cafe of 385, 512
Poultry, on a difeafe among 455
Polypus antri highmori, remarkable cafe of *(with an engraving)* 6
Pork predifpofes to mortification 484
Powell's, Dr. lectures announced 285
Practical obfervations on fcarlet fever 25
Preventive medical inftitution, propofed report of 479
Prickly-glafs wort, defcription of 229
Primrofe, botanical defcription of 126
Prifoners at Norman-Crofs, treatment of 345
Prize queftion at Paris 96
Prophylaxis, on vaccine 209
Puerperal fever, on 27
Pulley's, Mr. cafe of dropfy 249

Quacks and empiricifm, letters on 137, 212, 423
——— advertifements of 138
Queries on vaccination, anfwers to 11
Queftion, Paris prize 96

Rabies canina, obfervations on 513
Rampions, botanical defcription of 129
Rafpatory, defcription of an improved 202
Rattle-fnake, plantain faid to be a cure for the bite of the 30
Reading, remarkable chalybeate fpring at 240
Regnault, Dr. on the lichen iflandicus 463
Reid's, Dr. lectures announced 384
Report of the committee of the Houfe of Commons on the fever inftitution 161
Report of a Medical Committee 573
Richard's, Meff. analyfis of broth from bones 353
Ring, Mr. on the ufe of fpongia ufta 8
——— on diarrhœa and cholera morbus 102
——— obfervations on 219
——— review of his anfwer to Mr. Goldfon 181
——— his reply to the reviewer 282
——— on the London Difpenfatory, &c. 356
——— on eruptions after vaccination 358
——— on the cafe at Kenfington 446, 529
——— on the diffufion of vacciolation 447
——— on Dr. Rollo's cafes of inoculation 531
Roberts's, Dr. lectures announced 285
Rodman's, Dr. cafe of aneurifm of the aorta 235
Rollo's, Dr. cafes of inoculation reviewed 263
——— obfervations on 531
Roffi's, M. cafes of Galvanifm in hydrophobia 259
——— remarks on 387, 513
Rowley's Schola Medicinæ reviewed 469
Rupture-wort, fmooth, defcription of 227

Salivary Glands in animals, on 548
Samphire, defcription of rock 363
Saunders's Mr. propofals for a new Difpenfary 576
Saxifrage, great burnet, defcription of 556
Scarlatina, on 537
Scarlet fever, practical obfervations on 25
Schaub's, Mr. experiments on charcoal 283
Schmidt's, Mr. mode of preparing ammoniated iron 192
Scott, Dr. his journey in Greece 448
Scottifh lovage, defcription of 364

Scirrhous tumours, obfervations on 75
Scrotum, cafe of excrefcence on the 204
Scurvy-grafs, botanical defcription of 129
Sea buckthorn, botanical defcription of 33
Semi-offeous tumour, cafe of *(with a plate)* 465
Sheep, effects of vaccination in preferving 93
——— epidemic diforder among 456
——— pock, on the 458
Shells, natural hiftory of Britifh, announced 96
Smallage, defcription of 556
Small-pox, faid to fucceed vacciolation 384
——— after vaccination, cafes of 79
——— on a fecond infection of 329
Smith's, Mr. cafe of wounded brachial artery 339
Smyth's effay on the flexible metallic bougies 276
Snow, Mr. on the cure of agues 340
Soda, the muriat of, its virtues in deftroying worms 383
Sore throat, practical obfervations on 25
Spalding's, Dr. bill of mortality in America 94, 190
Sphacelus, on 483
Spignel, botanical defcription of 368
Spindle-tree, botanical defcription of 222
Spirituous liquors, effects of drinking of 337
Spongia ufta, on the ufe of 9
Sponge, its ufe in ophthalmia 233
Squire's, Dr lectures announced 287
Steam apparatus, account of a *(with a plate)* 516
Stenhoufe, Dr. on the gout 66
Strangulated hernia, cafe of 337
Sudden death, cafe of 391
Sulphur-wort, description of 363
Suppuration, cafe of extenfive 252
Surgery, cafes in 513
Surgical inftruments, Mr. Jardine's improved 148
——— Dr. Clark's improved 554
——— obfervations, review of 176, 266
Suffex Jennerian Inftitution, proceedings of 10, 477
Swediaur's Dr. pharmacopœia, reviewed 565
Swedifh foffil, chemical analyfis of a 69
Sweetgale, botanical defcription of 36
Sydenham's liquid laudanum, its efficacy in intermittents 4
Syer's, Mr. lectures announced 288
Syphilis, on difeafes refembling 268, 504

Tartar, new method of preparing emetic 189

Tavares, Dr. on Peruvian bark in the gout 142

Taunton's, Mr. lectures announced 288

Temperature, on 341

Tefticles, on an atrophy of the 518

Thackeray, Mr. on variolous inoculation 331

——— on cold water in convulfions 508

Thomas's St. Hofpital, lectures at 284

———, Mr. lectures announced 285, 384

Thorn-apple, defcription of 131

Thynne's, Dr. lectures announced 285

Tourniquet, account of Mr. Blake's *(with a plate)* 336

———, defcription of Dr. Clark's 544

Trephine, defcription of an improved 200

Trevor, Mr. on the fheep-pock 461

Trotter's, Dr. effay on drunkennefs reviewed 86

——— cafe of gaftrodynia 289

——— on cold water in the gout 232

——— Effay, a new edition announced 576

Trye, Mr. on the cow pox inoculation 395

Tumours, on the claffification of 176, 266

Turton's, Dr. Medical Gloffary, reviewed 565

——— cafe of femiofleous 459

Typhus, on mercurial inunction in 52, 481

——— cured by yeaft 192

——— on the animal heat in 244

——— in London, ftate of 572

Vaccævaration, on 517

Vaccination, a permanent prefervative 11

——— examination of failures in 44

——— after vaccination, review of 79

——— its effects on fheep 93

——— Dr. De Carro on 122

——— Mr. Leefe on 164

——— on eruptions after 358

——— ftate of in Italy 283

——— on the diffufion of 447

——— on Dr. Rollo's cafes of 534

——— in New South Wales 537

Vaccine Pock Inftitution, notice from 90

——— veficle, on the modifications of the 99

——— prophylaxis on 209

——— inoculation, obfervations on 398

Vacciola, on the efficacy of 205, 207

——— on the matter of 241

Vacciolation, on 438

——— cafe of 467

——— experiments in 473

Vagina, cafe of imperforated 236

Vegetable acid, difcovery of a new 92

——— matter, prize queftion 96

——— fungus, cafe of poifon from 335, 512

Veins of bones, on the 551

Venereal difeafe, obfervations on 279

### INDEX.

Veterinary medicine, review of a syftem of 278

Villeneuve, his botanical work announced 192

Vinegar, new method of obtaining concentrated 111

Violet, botanical defcription of 223

Vitriol, white, its effects on a horfe *(note)* 278

Uwins, Dr. on the progrefs of medicine 419

Wales (New South) practice of vaccination in 537
Walker's, Dr. examination of failures in vaccination 44
——— Mr. Goldfon's reply to 49
——— on Mr. Goldfon's pamphlet 168
——— obfervations on vacciola 241
——— on Dr. Adams's obfervations on the cow-pox 333
——— on vacciolation 438
——— teftimonials of 443
——— cafe of vacciolation 467
——— on vaccination 540
Water, apparatus for filtering 96
——— cold, in convulfions, on 508
——— in gout, on 237
Water hemlock, defcription of 367
——— its efficacy in head ache 1
Wayfaring tree, botanical defcription of 557
Weftfield, Mr. on the fheep pock 459
Willes on a new medicine for the gout, reviewed 181
White's Syftem of Veterinary Medicine, reviewed 278
Whitlam, Mr. on the management of leeches 349
Wilkinfon, Mr. on the cortex falicis latifolia 372
——— on the management of leeches 485
Willan, Dr. on cutaneous difeafes 97
Williams's, Mr. fteam apparatus, account of *(with a plate)* 516
Wilfon's, Mr. lectures announced 285
Wind, how to meafure the ftrength of 383
Worm, obfervations on the Guinea 434
Worms in the inteftinal canal, how to deftroy 383
Wounds, review of a Treatife on Gun-fhot 174

Yeaft, its efficacy in typhus 192

### REFERENCES TO THE PLATES.

Remarkable Case of a Polypus — — — — — — 6
Longitudinal Section of the Rectum, &c. — — — — *ib.*
Mr. Jardine's improved Trephine and Raspatory — — 198
Horny Excrescence on the Scrotum — — — — — *ib.*
Mr. Blake's Screw Tourniquet — — — — — — 336
Dr. Patterson's Case — — — — — — — — *ib.*
Mr. Greenhow's Case of Caries of the Os Frontis — — 394
Mr. Leeson's Case of Semiosseous Tumour — — — 465
Mr. Brody's Case of Exfoliation — — — — — — 513
Mr. Williams's Steam Apparatus — — — — — — 516
Dr. Clark's improved Surgical Instruments — — — — 554

